# Rivermead assessment of somatosensory performance: Italian normative data

**DOI:** 10.1007/s10072-021-05210-5

**Published:** 2021-03-30

**Authors:** Cristina Russo, Viviana Spandri, Marcello Gallucci, Peter Halligan, Nadia Bolognini, Giuseppe Vallar

**Affiliations:** 1 Department of Psychology and Milan Center for Neuroscience-NeuroMi, University of Milano-Bicocca, Piazza dell’Ateneo Nuovo 1, Building U6, 20126 Milan, Italy; 2 Neurology-Stroke Unit, Manzoni Hospital, Lecco, Italy; 3 School of Psychology, Cardiff University, Cardiff, UK; 4 Laboratory of Neuropsychology, IRCCS Istituto Auxologico Italiano, Milan, Italy

**Keywords:** Rivermead assessment of somatosensory performance, RASP, Normative data, Somatic sensation

## Abstract

The Rivermead assessment of somatosensory performance (RASP) provides a quantitative assessment of somatosensory processing, suitable for brain-damaged patients suffering from stroke. It consists of seven subcomponents: Subtest 1 (sharp/dull discrimination), Subtest 2 (surface pressure touch), Subtest 3 (surface localization), Subtest 4 (sensory extinction), Subtest 5 (2-point discrimination), Subtest 6 (temperature discrimination), and Subtest 7 (proprioception). Overall, the RASP assesses 5 bilateral body regions: face (cheek), hand (palm and back), and foot (sole and back). This study aimed at providing normative data and cut-off scores for RASP subtests, for each body region, in a large Italian population sample. We present results from 300 healthy Italian individuals aged 19 to 98 years. Data represent a comprehensive set of norms that cover each subtest and each body region tested. Performance in Subtests 1, 5, and 6 decreased, for some body regions, with increasing age. Based on these results, norms were stratified for age (seven groups), with the pathological/non-pathological cut-off coinciding with the 5th percentile. Conversely, other results were not influenced by age; in such cases, a single error, in each body region, has to be considered indicative of pathological performance. This independent investigation of all subcomponents of the somatosensory system, for each body region, further confirms RASP’s potential in clinical practice, for neurological assessment, as well as in research settings.

## Introduction

The examination of somatic sensation requires the assessment of a range of different processes, including tactile detection, acuity, pain, proprioception, vibration, temperature sensitivity, and stereognosis [1]. Somatosensory dysfunctions occur in many neurological conditions, affecting both the central and the peripheral nervous system [2]. In stroke, about 6 out of 10 survivors suffer from some somatosensory impairment [3], with different incidence among studies [4] due to various factors, as severity and level of impairment [5]. Another important source of variability is related to the affected body region [4]. Noteworthy, in clinical practice, somatic sensation is commonly dichotomously determined as being “present” or “absent” [5].

Somatosensory deficits are considered a negative predictor of functional outcome after stroke [6]: their presence is associated with motor activity limitations and a longer length of stay in hospital or nursing home [7]. Furthermore, somatic sensation is functionally interconnected with motor performance [8]; hence, it largely influences motor recovery after stroke [9]. Somatosensory deficits after unilateral stroke primarily affect the side of the body contralateral to the hemispheric lesion (contralesional), sometimes extending to the ipsilesional side [5, 10, 11].

In clinical practice, the main standardized tools available include the *Fugl Meyer Sensory Scale* — FMSs [12], the *Nottingham Sensory Assessment* — NSA [13], the *Quantitative Sensory Testing* — QST [14], and the *Rivermead Assessment of Somatosensory Performance* — RASP [15]; for reviews, see [4, 16].

The RASP [15] developed at the Rivermead Rehabilitation Center in Oxford provides a quantitative examination of 5 bilateral body parts: face (cheek), hand (palm and back), and foot (sole and back). The RASP comprises seven subtests, divided into five “primary” (sharp/dull discrimination, surface pressure touch, surface localization, temperature discrimination, and movement and direction proprioception) and two “secondary” (extinction and 2-point discrimination) ones. The RASP features a precise and validated protocol and shows good psychometric properties: test-retest (*r* = .92), inter-tester (*r* = .92) reliability, and validity (*s* = .52–.54). Moreover, the RASP can be used to assess somatosensory performance of patients in the sub-acute stage after stroke, providing information regarding deficits and recovery of somatosensory functions [9]. The RASP was first validated in UK in a sample of 100 post-stroke brain-damaged patients and in 50 age-matched healthy participants [15] and then in Germany in 60 stroke patients, showing a good-to-excellent inter-rater reliability across subtests [17]. The battery has the advantage of being short (the assessment lasts about 25–35 min), easy to administer, and simple to score [18]. The use of the RASP has been also recommended, due to its interval scales, for statistical analyses [4].

The present study provides RASP normative data in a sample of Italian healthy participants.

## Materials and methods

### Participants

Three-hundred Italian, neurologically healthy, individuals, with no history or evidence of neurological or psychiatric disorders, entered this study. The sample comprised 124 males (M) and 176 females (F), with a mean age of 50.8, standard deviation (SD) = ±21.1 years (M = 53.1 ± 20, F = 49.2 ± 22), and a mean schooling of 12.8 ± 4.3 years (M = 13 ± 4, F = 12.7 ± 4). Following a standardized interview [19], six participants were classified as left-handed (2% of the total), 2 ambidextrous (0.07%), and 298 (97.3%) right-handed. The study conformed to Declaration of Helsinki and was approved by the ethical committee of the IRCCS Istituto Auxologico Italiano.

### Procedure

#### RASP tools

**Fig. 1:**
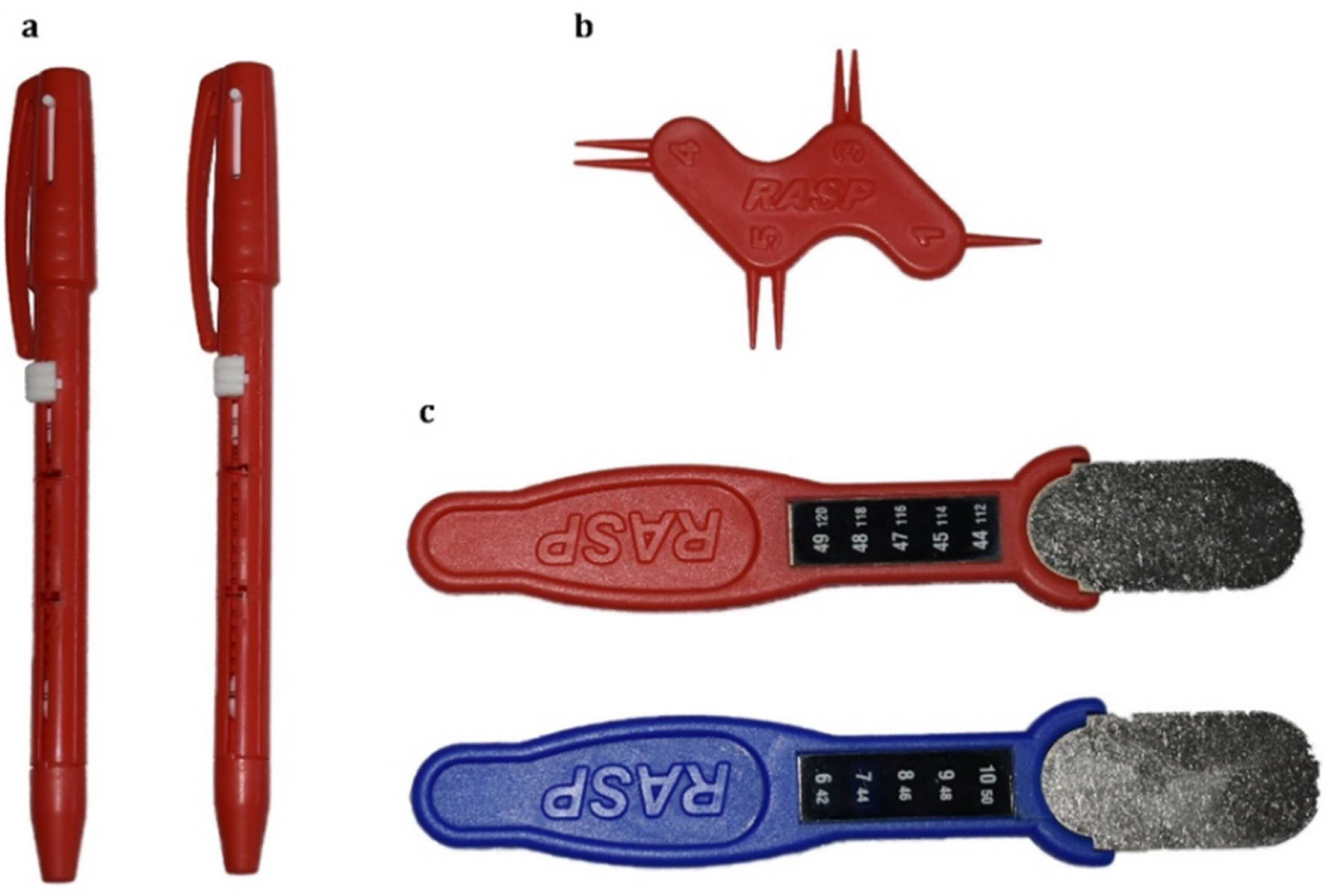
RASP instruments. a Neurometer: a pen-shaped device that allows two amounts of pressure (i.e., 15.5 or 67.5 g) to be applied to the target body area. On its superior top, a single-use Neurotip (sharp or dull) could be inserted; b Neurodisc: 2-point discriminator with fixed distances of 3, 4, and 5 mm; there is also a single-point tip; c Neurotemp: LCD temperature devices

The RASP kit comprises three bespoke instruments: the *Neurometer*, the *Neurotemp*, and the *Neurodisc*, shown in Fig. 1. The Neurometer is a pen-shaped device that allows a given amount of pressure to be applied to an area. This instrument provides two different amounts of pressure (i.e., 15.5 or 67.5 g). On the top, a sterile single-use pin, called a *Neurotip*, can be inserted. *Neurotips* are either sharp or dull. The RASP kit provided 2 Neurometers in order to allow a double stimulation (see below). The *Neurodisc* is the tool used for testing 2-point discrimination, with three distances: 3, 4, and 5 mm.

The *Neurotemps* (1 red and 1 blue) are simple temperature devices with liquid crystal display (LCD). The red *Neurotemp* is used to deliver a “warm” stimulation, of about 44–49 °C, brought by immerging the instrument in boiled water for around 30 s. The blue *Neurotemp*, after immersion in ice water for about 30 s, delivers a “cold” stimulation, of about 6–10 °C. Before testing, RASP tools are shown to the participant, along with a brief description of each subtest.

#### RASP subtests

The RASP was administered following the original instructions [15, 18], translated into Italian. The RASP comprised seven subtests, delivered to participants, who had received instructions to keep their eyes closed throughout the testing session. In each task, participants were encouraged not to worry if they did not feel a given stimulus, rather just to do their best to perform the task. The number of correct responses, for each region, was manually recorded on the scoring sheet. In the following, a brief description of each subtest is provided; stimulus order and randomization followed the original protocol [18].

##### Subtest 1: sharp/dull discrimination

Two *Neurometers* were used. On each *Neurometer*, a sterile, single-use *Neurotip* (one with a sharp and one with a dull end) was inserted. For this subtest, the sharp or dull stimulus was applied on each test region. Participants were instructed to report, for each trial, which kind of stimulation (“sharp” or “dull”) was felt. For each region, eight stimuli were presented: three dull (D), three sharp (S), and two sham (§) trials in the following order: S-§-D-D-S-S-§-D. In sham trials, the *Neurometer* was applied nearby the body (within 15 cm), emitting the same audible sound of the test stimulus, but avoiding any contact with the participant’s skin. Sixty trials (30 left, 30 right) and 20 § stimuli (10 left, 10 right) were administered to ten body regions: right and left face, right and left hand (palm and back), and right and left foot (sole and back).

##### Subtest 2: surface pressure touch

This subtest was administered using one *Neurometer* set to deliver a 15.5 g pressure. A series of single stimuli was applied, in a real (touch) or sham (non-touch) fashion, on each test region. The order and the type (touch or non-touch) of the stimulations were pseudo-randomized as in the original protocol. Sham trials consisted in non-touch stimuli applied as in Subtest 1. Participants were instructed to report, in each trial, whether or not they had felt the stimulation. For each region, six touch (T) and two sham (§) trials were delivered in the following order: T-§-T-T-T-T-§-T. Sixty trials (30 left, 30 right) and 20 § stimuli (10 left, 10 right) were administered to ten body regions: right and left face, right and left hand (palm and back), and right and left foot (sole and back).

##### Subtest 3: surface localization

This subtest required one *Neurometer*, set to deliver a 15.5 g pressure. Participants had to report, in each trial, on which body side (right or left) the stimulus had been applied. Sixty stimuli (30 left and 30 right) were administered to the face, palm and back hand, and sole and back foot. For each body area, 12 stimuli were presented (6 for each side), in a pseudo-randomized order.

##### Subtest 4: sensory extinction

Both *Neurometers* were used, set to a 67.5 g pressure. Participants were instructed to report, in each trial, on which body side (right or left or both) the stimulus was applied. Following a pseudo-randomized order, the evaluator applied the stimulus on the right, on the left, or on both sides, simultaneously, of the following regions: face and hands (on the back). For each region, six bilateral (B) and two unilateral (one left, L, and one right, R) stimuli were presented in the following order: B-L-B-B-B-B-R-B. For the two body parts, 12 bilateral and four single trials were given.

##### Subtest 5: 2-point discrimination

The *Neurodisc* was applied on the fingertip of the left and of the right index finger. In a pseudo-randomized order, the evaluator depressed the participant’s skin for approximately 1 mm before releasing, with either a single- or a 2-point tip. Participants were instructed to report which kind of stimulation (“single” or “double”) they had felt. On each index finger, eight trials were delivered: two single-point trials (1) and six 2-points trials (2) in the following order 2-1-2-2-2-2-1-2. The subtest started with the 2-point distance of 3 mm. If the participant was able to correctly report the 2 points in at least four out of six trials, the test was considered successfully completed and the 2-point discrimination threshold was considered to be 3 mm. If this was not the case, the test was repeated with a 4 mm distance; if the participant was able to correctly report the 2 points at least four out of six times, the threshold was considered to be 4 mm. If this was not the case, the test was repeated with the 5 mm 2-point distance; if the participant failed also at this distance, the subtest was considered failed.

##### Subtest 6: temperature discrimination

Both *Neurotemps* were used. Participants had to report which kind of stimulation, “warm” or “cold,” they felt. Sixty trials were administered to the 10 body regions: right and left face, right and left hand (palm and back), and right and left foot (plant and back). For each region, six stimuli were presented, three warm (W) and three cold (C), in the following order: W-C-C-W-W-C.

##### Subtest 7: joint movement and movement direction discrimination

The evaluator moved a participant’s joint either up or down in a pseudo-randomized sequence. Participants had to report whether they felt the movement (“Joint movement”) and in which direction (“up” or “down,” i.e., “Movement direction discrimination”). The score was the number of passive movements correctly reported (7a, “Joint movement”), and their direction (7b, “Movement direction discrimination”). The tested joints were in the right and left upper (elbow, wrist, and thumb) and lower (ankle and big toe) limbs; each joint was moved six times in the following order: up-down-down-up-up-down.

### Statistical analysis

Statistical analyses were performed through IBM® SPSS® Statistics, version 25.0.

For each subtest (except Subtest 5, 2-point discrimination), the number of correct responses for each body region was considered. Specifically:

- *Subtest 1 (sharp/dull discrimination)*, *Subtest 2 (surface pressure touch)*, *Subtest 3 (surface localization)*, and *Subtest 6 (temperature discrimination)*: right and left face, right and left hand (palm and back), and right and left foot (plant and back);
- *Subtest 4* (sensory extinction): face and (back) hands;
- *Subtest 7a (joint movement)* and *Subtest 7b (movement direction discrimination)*: right and left superior limbs (elbow, wrist, and thumb); right and left inferior limbs (ankle and big toe).

With respect to *Subtest 5* (2-point discrimination), since a pass or fail scoring procedure was used, the outcome of passing (3 mm, 4 mm, 5 mm) or failing the subtest was scored.

To assess the role of demographic variables, each subtest was submitted to a Poisson linear model considering age, gender, and schooling, both independently and in interaction. Significant threshold was set at alpha = 0.05.

Scores in sham trials, which detect false positives, were not analyzed statistically, because no participant gave this kind of response in any task.

The *Age* variable was divided as follows: 1: ≤ 25 years (*N* = 42); 2: between 26 and 35 years (*N* = 47); 3: between 36 and 45 years (*N* = 43); 4: between 46 and 55 years (*N* = 40); 5: between 56 and 65 years (*N* = 36); 6: between 66 and 75 years (*N* = 44); 7: ≥ 76 years (*N* = 45).

The *Schooling* variable was divided as follows: 1: ≤ 5 years (i.e., elementary school, *N* = 30); 2: between 6 and 8 years (i.e., middle school, *N* = 50); 3: between 9 and 13 years (i.e., college, *N* = 111); 4: between 14 and 16 years (i.e., bachelor degree, *N* = 31); 5: ≥ 17 years (i.e., master degree, further specialization, or PhD, *N* = 78).

With respect to *Gender*, the sample was divided as follows: 1: male (*N* = 124); 2: female: (*N* = 176).

## Results

For each subtest, and for each body region, chi-square (χ^2^) and *P*-values were calculated and a significant effect of *Age* on performance was found. Specifically, the mean correct responses, and the values corresponding to the 5th percentile for each body region, at different levels of the factor *Age* are provided. The pathological/non-pathological cut-off coincides with the 5th percentile. It should be noted that only the *Age* factor was considered, albeit the distribution of participants within *Age* and *Schooling* was not entirely independent (the two factors were correlated, with elderly participants presenting with lower schooling, and vice-versa); this option was adopted as *Schooling* was not expected to affect somatic sensation. The absence of significant *Age* × *Schooling* interactions (all *P*s > 0.05) confirmed this prediction.

Conversely, in case of a non-significant effect of *Age*, the values of the mean correct responses were reported corresponding to the cut-offs, rounded to the nearest whole number.

With respect to Subtest 5, percentages of participants who successfully completed or failed the task, and the minimum distance at which they could perceive the 2 points as different, for each *Age* category, are presented. Then, the mean score and the value corresponding to the 5th percentile for each index finger are provided.

### Subtest 1: sharp/dull discrimination

The Poisson linear model revealed a significant effect of *Age* with respect to the right [χ^2^_(6)_ = 13.87, *P* = 0.031] and the left [χ^2^_(6)_ = 28.66, *P <* 0.001] foot. Also, *Schooling* was significant for the same body regions: right [χ^2^_(4)_ = 12.68, *P* = 0.013] and left [χ^2^_(6)_ = 28.66, *P* < 0.001] foot, respectively. As no interaction *Age* × *Schooling* [*P* = 0.9] was found, in Table 1, values considering the variable *Age only* are reported.

**Table 1:** Mean score, 5th percentile, and cut-off for Subtest 1, regions: right and left foot

	Subtest 1: sharp/dull discrimination					
	Right foot			Left foot		
Age	Mean score (max = 12)	5th percentile	Cut-off	Mean score (max = 12)	5th percentile	Cut-off
≤ 25	11.23	9.00	9	11.19	9.00	9
26–35	10.85	9.00	9	10.83	9.00	9
36–45	10.93	8.00	8	10.51	7.20	7
46–55	10.70	8.00	8	10.25	7.05	7
56–65	10.50	7.00	7	9.61	7.00	7
66–75	9.82	6.25	6	9.14	5.25	5
≥ 76	9.13	6.00	6	8.27	5.00	5

For all the other body regions (i.e., right and left face, right and left hand) mean correct responses are reported in Table 2. This value was considered the cut-off.

**Table 2:** Top: mean score and cut-off for Subtests 1 (sharp/dull discrimination), 2 (surface pressure touch), 3 (surface localization), and 6 (temperature discrimination), regions: right and left face, right and left hand, right and left foot. Middle: mean score and cut-off for Subtest 4 (sensory extinction), regions: face and hands. Bottom: mean score and cut-off for Subtests 7a (joint movement) and 7b (movement direction discrimination), regions: right and left upper limb (UL), right and left lower limb (LL)

	Right face		Left face		Right hand		Left hand		Right foot		Left foot	
	Mean score	Cut-off	Mean score	Cut-off	Mean score	Cut-off	Mean score	Cut-off	Mean score	Cut-off	Mean score	Cut-off
S1	5.8	6	5.8	6	11.7	12	11.5	12	See Table 1		See Table 1	
S2	6.00	6	6.00	6	12.00	12	12.00	12	12.00	12	12.00	12
S3	6.00	6	6.00	6	12.00	12	12.00	12	12.00	12	11.9	12
S6	6.00	6	5.9	6	11.9	12	11.8	12	11.2	11	See Table 5	
S4	Face				Hands							
	6.00	6			6.00	6						
	Right UL		Left UL		Right LL		Left LL					
S7a	18.00	18	18.00	18	12.00	12	12.00	12				
S7b	17.9	18	17.9	18	11.9	12	11.9	12				

### Subtests 2–3–4: surface pressure touch, surface localization, sensory extinction

For each subtest, none of the models reached significance (all *P*s < 0.05). Table 2 reports the values of the mean correct responses and the corresponding cut-off value: a single error, in each body region, was considered indicative of pathological performance.

### Subtest 5: 2-point discrimination

The percentages of participants, who passed the subtest, are reported in Table 3 for the right and left index fingers.

**Table 3:** Percentages of participants who passed or failed the subtest (i.e., > 4 double stimuli) in each condition (i.e., 3, 4, and 5 mm) for the right and for the left index finger

Subtest 5: 2-point discrimination								
Right index finger					Left index finger			
Age	3 mm	4 mm	5 mm	Failed	3 mm	4 mm	5 mm	Failed
≤ 25	93.0%	4.7%	2.3%	0.0%	88.4%	9.3%	2.3%	0.0%
26–35	66.0%	25.5%	8.5%	0.0%	63.8%	23.4%	12.8%	0.0%
36–45	58.1%	20.9%	20.9%	0.0%	53.5%	20.9%	25.6%	0.0%
46–55	47.5%	20.0%	32.5%	0.0%	40.0%	27.5%	32.5%	0.0%
56–65	42.1%	26.3%	31.6%	0.0%	39.5%	15.8%	44.7%	0.0%
66–75	47.7%	15.9%	34.1%	2.3%	29.5%	25.0%	45.5%	0.0%
≥ 76	26.7%	15.6%	46.7%	11.1%	28.9%	13.3%	51.1%	6.7%

In order to determine normative values, data were re-coded as follows: 3 = successful (at least 4 out of 6 correct responses) 2-point discrimination at 3 mm; 2 = successful 2-point discrimination (at least 4 out of 6) at 4 mm; 1 = 2-point successful discrimination (at least 4 out of 6) at 5 mm; 0 = failure, less than 4 (out of 6) accurate 2-point discrimination at 5 mm.

The Poisson linear model revealed a significant effect of *Age* for both the left [χ^2^_(6)_ = 19.789, *P* = 0.003] and the right [χ^2^_(6)_ = 21.072, *P* = 0.002] index. In addition, *Schooling* was significant for the left [χ^2^_(6)_ = 9.656, *P* = 0.047] index, only. As no interaction Age × Schooling [*P* = 1] was found, Table 4 reports the mean correct responses, the 5th percentile, and the corresponding cut-off value (i.e., maximal distance to allow at least 4 out of 6 double stimuli), considering the variable *Age*.

**Table 4:** Mean score, 5th percentile, and cut-off for Subtest 5, regions: right and left index finger

Subtest 5: 2-point discrimination						
	Right index finger			Left index finger		
Age	Mean score (range 0–3)	5th percentile	Cut-off	Mean score (range 0–3)	5th percentile	Cut-off
≤ 25	2.91	2	4 mm	2.86	2	4 mm
26–35	2.57	1	5 mm	2.43	1	5 mm
36–45	2.37	1	5 mm	2.28	1	5 mm
46–55	2.15	1	5 mm	2.07	1	5 mm
56–65	2.11	1	5 mm	1.95	1	5 mm
66–75	2.09	1	5 mm	1.84	1	5 mm
≥ 76	1.58	0	-	1.64	0	-

### Subtest 6: temperature discrimination

The Poisson linear model revealed an effect of *Age* for the left foot [χ^2^_(6)_ = 18.173, *P* = 0.006]. Table 5 reports the mean correct responses, the value corresponding to the 5th percentile, and the cut-off for the left foot considering the independent variable *Age*.

**Table 5:** Mean score, 5th percentile, and cut-off for Subtest 6, region: left foot

Subtest 6: temperature discrimination			
Left foot			
Age	Mean score (max = 12)	5th percentile	Cut-off
≤ 25	11.84	11.00	11
26–35	11.34	9.00	9
36–45	11.21	8.00	8
46–55	10.67	7.05	7
56–65	10.42	7.00	7
66–75	10.20	6.25	6
≥ 76	9.27	5.00	5

For all the other body regions (i.e., right and left face, right and left hand, right foot), the values of the mean correct responses, which corresponds to the cut-off value, are reported in Table 2.

### Subtests 7a–b: joint movement, movement direction discrimination

None of the models were significant (all *P*s < 0.05); a single error, in each body region, reflected a pathological performance. Results are reported in Table 2.

## Discussion

The aim of the present study was to collect normative data in a large Italian population for the RASP, providing normative values, along with cut-off scores, separately for body parts in all subtests. Validity, reliability, and test-retest and inter-rater variability had been evaluated in the original works, demonstrating good psychometric properties [15, 17].

Compared to the original study [15], the present study included a larger sample (*N* = 300) of neurological healthy participants (50 healthy controls in the original English version) and took into consideration the *Age* factor, provided cut-offs also for 2-point discrimination (Subtest 5), and scores for each tested body region.

In previous studies [15, 17], a single cut-off value was established for each subtest that did not consider potential sources of variation such as *Age*, *Gender*, and *Schooling.* The role of these variables was assessed in this study, both independently and in combination. Importantly, statistical analyses confirm a significant role of the factor *Age*, which influences performance in all the subtests assessing discrimination abilities: sharp/dull (Subtest 1), 2-point (Subtest 5), and temperature (Subtest 6). Progressive deterioration of somatosensory performance with advancing age has been reported by several investigators. For instance, tactile acuity has been shown to decrease with age, particularly over 60 years [20–22], as well as joint proprioception, which becomes less accurate [23, 24].

Conversely, neither *Gender* nor *Schooling* factors were found to affect somatosensory processing in healthy participants. The lack of gender effects is in keeping with previous evidence showing the absence of sex difference in the same sensory tasks used here, while it seems to influence more complex haptic tasks, but with some inconsistencies across studies [22, 25]. As regards schooling, no effects were expected, given the low-level sensory abilities assessed by RASP; in studies on haptic processing, putative effects of schooling are intermingled with those of development [25].

The present study also provides cut-off values, for both the left and the right index fingers and separately for age group, for 2-point discrimination (Subtest 5), not previously reported in the original work [15]. The availability of cut-off scores for this subtest is clinically useful when assessing body-specific somatosensory deficits in neurological disorders, such as stroke, but also for other diseases such as anorexia nervosa [26, 27] and cerebral palsy [28].

The original work [15] provided normative data from participants in the United Kingdom, also testing stroke patients with left (*N* = 50) and right (*N* = 50) cerebral hemispheric damage; results were reported separately for the contralesional and the ipsilesional sides of the body in stroke patients, but not in healthy participants, for which normative data referred to the sum of all five regions tested (face, palm and back of the hand, sole and back of the foot).

However, unilateral focal cerebral lesions may bring about body-part-specific somatosensory impairments. For instance, impairments of tactile localization have been shown to occur in more than the 50% of stroke survivors at the wrist level, but rarely present at the contralesional side of the face (~7% of incidence) [10]. Differences in proprioception and tactile processing were reported for arms and legs, with the lower limb being more impaired [29]. Indeed, the lower and upper limbs tend to follow different trajectories of recovery, with more improvement in upper-limb than in lower-limb sensation [10]. As a consequence, it is clinically important to have body-part-specific normative data that can be used in prospective observational studies, largely neglected in post-stroke management [29]. Moreover, the availability of unique cut-offs for each subtest also permits their administration to a given body region in isolation, thus avoiding the delivering of subtests, considered less relevant by the clinician, to some patients, all of which can be time-consuming and uninformative [10].

In conclusion, the present study provides the first Italian normative data for the RASP and cut-off values that can be used to identify somatosensory impairments. The study confirmed the RASP as a valid tool capable of measuring several different aspects of somatosensory sensation. Standardized and completed with normative values, the RASP provides clinicians and researchers with a valid instrument for both assessment and follow up protocols.

### Availability of data and material

The data that support the findings of this study are available from the corresponding author upon reasonable request.

### Code availability

Not applicable.
